# Congenital syphilis related to primary health care and prenatal care coverage: a spatial analysis, Minas Gerais, 2020-2022

**DOI:** 10.1590/S2237-96222025v34e20240495.en

**Published:** 2025-07-11

**Authors:** Bruna Betiatti Benatatti Eller, Marcelle Aparecida de Barros Junqueira, Lucio Borges de Araújo

**Affiliations:** 1 Universidade Federal de Uberlândia, Programa de Pós-Graduação em Saúde da Família, Uberlândia, MG, Brazil; 2 Universidade Federal de Uberlândia, Instituto de Matemática e Estatística, Uberlândia, MG, Brazil

**Keywords:** Syphilis, Congenital, Prenatal Care, Primary Health Care, National Health Programs, Health Information Systems, Sífilis Congénita, Atención Prenatal, Atención Primaria de Salud, Programas Nacionales de Salud, Sistemas de Información en Salud

## Abstract

**Objective:**

To analyze variation in congenital syphilis incidence, according to prenatal indicators and primary health care (PHC) coverage in the state of Minas Gerais, between 2020 and 2022.

**Methods:**

Spatial analysis presented by means of clusters, using the non-hierarchical k-means technique and spatial georeferencing, with secondary data from the Primary Care Health Information System and the Minas Gerais State Health Department Health Surveillance Portal.

**Results:**

Cluster 1 presented lower prenatal indicators (35.5%) and PHC coverage (88.6%), with high congenital syphilis incidence (36.5 cases/1,000 live births). Cluster 2 demonstrated lower incidence of the disease (5.1 cases/1,000 live births) and better PHC (96.5%) and prenatal (57.5%) coverage. Cluster 3 recorded prenatal indicators with low performance (25.7%), adequate PHC coverage (92.2%), and low congenital syphilis incidence (3.21 cases/1,000 live births).

**Conclusion:**

Different care behaviors and congenital syphilis incidence were identified between health macro-regions and/or municipalities of Minas Gerais; as such, the study can contribute to the formulation of public policies to address this infection.

## Resumen

**Objetivo:**

Analizar la variación de la incidencia de sífilis congénita, según indicadores prenatales y cobertura de atención primaria de salud (APS) en el estado de Minas Gerais, entre 2020 y 2022.

**Métodos:**

Análisis espacial presentado a través de conglomerados, utilizando la técnica k-medias no jerárquicas y georreferenciación espacial, con datos secundarios del Sistema de Información en Salud para Atención Primaria y del Portal de Vigilancia en Salud de la Secretaría de Estado de Salud de Minas Gerais.

**Resultados:**

El conglomerado 1 presentó menores indicadores prenatales (35,5%) y cobertura de APS (88,6%), con alta incidencia de sífilis congénita (36,5 casos/1.000 nacidos vivos). El conglomerado 2 mostró una menor incidencia de la enfermedad (5,1 casos/1.000 nacidos vivos) y una mejor cobertura de APS (96,5%) y prenatal (57,5%). El conglomerado 3 registró bajo desempeño en indicadores prenatales (25,7%), adecuada cobertura de APS (92,2%) e incidencia reducida de sífilis congénita (3,21 casos/1.000 nacidos vivos).

**Conclusión:**

Se identificaron diferentes comportamientos de atención e incidencia de sífilis congénita entre macrorregiones sanitarias y/o municipios de Minas Gerais; de esta forma, el estudio puede contribuir a la formulación de políticas públicas para combatir esta infección.

**Ethical aspects**

This research used public domain anonymized databases.:

## Introduction

Syphilis is a sexually transmitted infection that, during pregnancy, can cause an increase in the proportion of fetal deaths by up to 40.0%, in addition to neonatal deaths. Transmission from mother to fetus can be reduced by up to 98.0% if syphilis is diagnosed and treated in pregnant women in a timely manner (1).

The epidemiological scenario of congenital syphilis, on a global scale, reached estimates of 473 (385-561)/100,000 live births in 2016, with emphasis on the African continent, which recorded 61.0% of total cases (2). In Brazil, in 2021, congenital syphilis incidence rates reached 9.9/1,000 live births (3).

The World Health Organization has developed strategies for the elimination of congenital syphilis, highlighting that the incidence rate should be 0.5 or fewer cases per 1,000 births by 2030 (4). The strategic role of primary health care (PHC) in prevention and immediate intervention in relation to gestational syphilis is fundamental, as it is the priority location for provision of prenatal care, expansion of diagnostic tests and timely treatment (5).

The quality of prenatal care remains a challenge in health services, where shortcomings in care are associated with the resurgence of this condition (6). Insufficient health coverage is also one of the pillars that easily contribute to structural barriers to access and interfere in prenatal care for pregnant women (7).

In 2019, a federal funding model was established, namely the *Previne Brasil* Program, with new methodological proposals for PHC action and evaluation. As such, indicators were assigned, in this new policy, to direct and evaluate the actions offered in PHC – including prenatal indicators, with the purpose of ensuring early care and continuous monitoring during the gestational period (8).

There is a considerable gap in studies that relate the quality of PHC care to congenital syphilis incidence, considering the functioning and organization of health service management between territories. This study aimed to analyze the variation in congenital syphilis incidence rates, according to the Previne Brasil prenatal care indicators and PHC coverage, in the municipalities of Minas Gerais, between 2020 and 2022.

## Methods

### Design

This study has a spatial, cross-sectional and analytical analysis design, using secondary data from public health information systems to analyze the relationship between prenatal care indicators and congenital syphilis incidence rates in municipalities of Minas Gerais, between 2020 and 2022.

### Setting

Secondary data from the main information systems of the Brazilian National Health System (*Sistema Único de Saúde* - SUS) were used, namely the Primary Care Health Information System and the Minas Gerais State Health Department Health Surveillance Portal, which are publicly accessible.

Minas Gerais has 28 Regional Health Superintendencies, distributed across 12 mesoregions, with a population of approximately 21 million inhabitants. The Metropolitan Region of Belo Horizonte, with over 5.5 million inhabitants, is the economic and political center of the state, with a strong presence of services, commerce and industry. The Central Mineira mesoregion, with 2.5 million inhabitants, has a diversified economy, with agriculture, livestock and mining.

The Norte de Minas and Vale do Rio Doce mesoregions have economies based on agriculture, livestock and mining, while the Triângulo Mineiro/Alto Paranaíba mesoregion is known for agribusiness. The Zona da Mata mesoregion is famous for its coffee production. The Campo das Vertentes, Vale do Mucuri, Jequitinhonha and Alto Jequitinhonha mesoregions present challenges in health infrastructure in rural areas, with aging and dispersed populations.

### Population data

Initially, data collection was performed by accessing information on congenital syphilis incidence, population coverage by PHC teams and prenatal indicators included in the Previne Brasil Program. This allowed the structuring of a consistent database to support the statistical analysis of this study (9,10).

### Variables

The annual congenital syphilis incidence rate, accessed via the Minas Gerais State Health Department Health Surveillance Portal, was the main variable of this research, being measured for each municipality through the number of new reported cases of congenital syphilis divided by the number of live births in the same year/location, multiplied by one thousand. The percentage results of the prenatal indicators formed the remaining variables, identified via the Primary Care Information System. Indicator 1 assesses the proportion of pregnant women with at least six prenatal consultations, with the first consultation taking place up to the 12^th^ week of pregnancy. In turn, indicator 2 measures the proportion of pregnant women undergoing tests for syphilis and human immunodeficiency virus (HIV).

### Data sources and measurement

In order to build the municipal scenarios with prenatal indicators, the means were calculated separately for indicator 1 and also for indicator 2, according to the results achieved in the first, second and third four-month periods of 2022 of the Previne Brasil Program. The parameters used to determine quality prenatal care were also recommended as a target for evaluating the performance of PHC teams, based on the Program, being 45.0% for indicator 1 and 60.0% for indicator 2. Population coverage by PHC teams, also included in this study as a variable, was expressed as annual average PHC coverage.

### Bias control

For the purposes of assessing PHC coverage in the municipalities studied, the average PHC coverage for Minas Gerais state was adopted as a reference. The calculation method is based on the number of people registered by the Family Health Strategy (FHS) and by PHC teams, in relation to the population estimated by the Brazilian Institute of Geography and Statistics (IBGE) (11).

Use of health information systems as a data source may have limitations, particularly in the context of health emergencies, such as the COVID-19 pandemic. The main challenges include underreporting, record inconsistencies, changes in care flows, and prioritization of serious cases. Additionally, health system overloading negatively impacted data quality. To minimize these biases, strategies such as cross-validation with secondary sources, analysis of temporal trends, statistical adjustments to correct underreporting, and exclusion of inconsistent data were implemented, thus ensuring the validity and reliability of the results obtained in this study.

### Study size

Data (congenital syphilis incidence rate and PHC indicator means) were extracted and processed in four-month periods for the years 2020, 2021 and 2022 for comparative temporal assessment, allowing a robust understanding of the epidemiological and healthcare scenario in Minas Gerais.

### Statistical methods

Cluster analysis was used when analyzing the data. This is a multivariate statistical approach that organizes distinct groups of municipalities based on their characteristics and similarities, considering the variables collected. The formation of these clusters combines municipalities with high internal homogeneity and high external heterogeneity, through the adoption of statistical techniques/functions with definitions of measures of proximity and distance between them, through Euclidean distance measurement (12).

The non-hierarchical k-means technique was applied, in which the elements are classified based on a previous definition of the number of clusters and the initial estimates of the centroids of each cluster. In order to avoid scale effects, the variables were standardized before forming the clusters.

### Data access and cleaning methods

Several cluster configurations were tested, and three clusters were chosen, considering theoretical interpretation and the ability to describe the components of each group. The municipality of Piau was identified as an atypical case, due to statistical dimensions that differed from the other records. After analysis, this case was excluded from the database used to form the clusters, in order to guarantee the representativeness and coherence of the variables studied. This preventive exclusion aims to ensure that the results of the cluster analysis are not influenced by atypical values, allowing a more accurate and reliable representation of the patterns and underlying structures in the data.

### Data pairing

The contribution of the variables to the formation of the clusters was assessed using the analysis of variance (ANOVA) technique. For each cluster, the mean, standard deviation and cluster frequency were estimated. The difference between clusters was considered statistically significant for each variable if it had a p-value>0.05. The analyses were performed using JAMOVI v. 2.3.28. In order to characterize and describe regional variability between the clusters, spatial georeferencing of the municipalities of Minas Gerais was developed using R v. 4.3.3., so that it was possible to identify on the map in which cluster each municipality had been allocated.

## Results

In the exploratory analysis, covering the period from 2020 to 2022, Minas Gerais revealed a peak incidence of congenital syphilis of 13.8 cases/1,000 live births in the second four-month period of 2022, and a 26.0% increase in cases, when compared to the values ​​recorded at the beginning and end of the four-month periods between 2020 and 2022. With regard to prenatal indicators 1 and 2, a positive evolution of the results was observed during the first four-month period of 2020 until the third four-month period of 2022.

**Figure 1:**
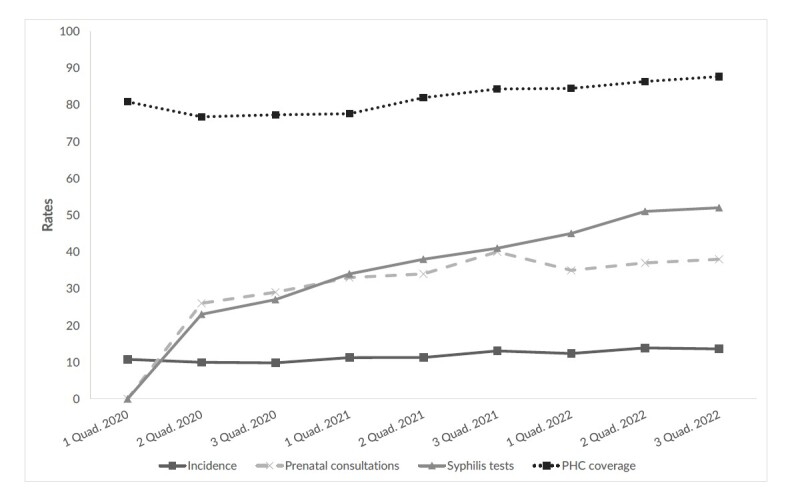
Congenital syphilis incidence, mean prenatal indicators and primary health care coverage, per 4-month period (quadrimester – Quad.). Minas Gerais, 2020-2022 (n=852)

Both indicators showed improvements, although in the third three-month period of 2021, the prenatal consultations indicator recorded a result of 40.0%, followed by a reduction in subsequent periods. In the third four-month period of 2022, the percentages achieved were 38.0% for indicator 1 and 52.0% for indicator 2. In the period from 2020 to 2022, Minas Gerais, in each four-month period, recorded an increase in the number of Family Health Strategy and PHC teams, with an annual average increase of 5.0%, culminating in 88.0% PHC coverage in the third four-month period of 2022 (Figure 1). 

**Figure 2:**
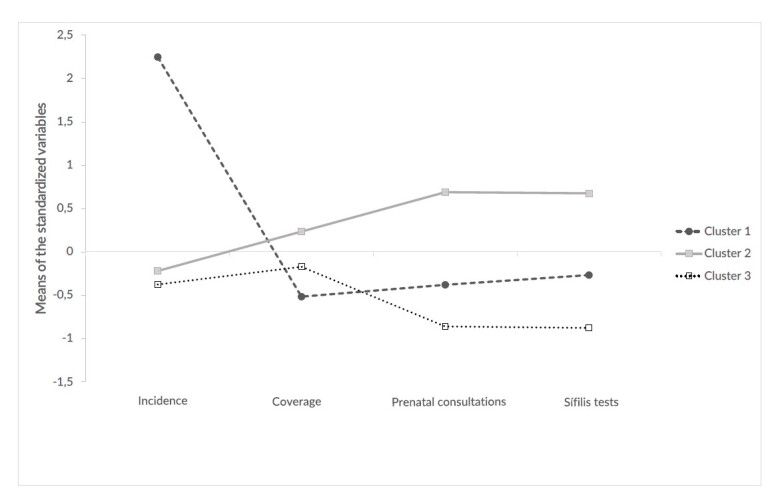
Mean congenital syphilis incidence rates, primary health care coverage, prenatal care indicator 1 (at least six consultations) and indicator 2 (testing for syphilis), by cluster. Minas Gerais, 2020-2022 (n=852)

In the cluster analysis model (Figure 2), in 2022, the configuration of the three clusters revealed distinctive characteristics, considering the combinations of the indicators analyzed, in which the profile of the municipalities was structured as follows:

- Cluster 1: composed of municipalities that showed simultaneous limitations, both in terms of low care performance related to prenatal and PHC coverage indicators, and in congenital syphilis incidence rates, which were high.
- Cluster 2: formed by municipalities with better PHC and prenatal coverage indicators, and lower congenital syphilis rates.
- Cluster 3: composed of municipalities that recorded low congenital syphilis incidence rates, but with lower prenatal indicators and high PHC coverage, compared to Cluster 2.

In detail (Table 1), Cluster 1 is represented by 96 municipalities, accounting for 11.2% of the total number of municipalities in Minas Gerais. Regarding congenital syphilis rates, it was the cluster with the highest mean incidence of congenital infection (36.5 cases/1,000 live births), higher than Clusters 2 and 3. In this cluster, the PHC team coverage rate (88.6%) was lower than for the other clusters.

**Table 1:** Number of municipalities, means (%), standard deviation (SD) of the congenital syphilis incidence rate, primary health care coverage, prenatal care indicator 1 (at least six consultations) and indicator 2 (testing for syphilis), by cluster. Minas Gerais, 2020-2022 (n=852)

Cluster	Number of municipalities	Congenital syphilis incidence rate % (SD)	Primary health care coverage % (SD)	Indicator 1 – prenatal consultations % (SD)	Indicator 2 – syphilis and HIV tests % (SDP)
1	96	36.5 (11.7)	88.6 (15.6)	35.5 (18.2)	50.1 (21.3)
2	444	5.1 (7.7)	96.5 (6.8)	57.5 (12.4)	72.6 (13.9)
3	312	3.2 (5.4)	92.2 (11.7)	25.7 (14.7)	35.5 (17.8)
p-value		<0.001	<0.001	<0.001	<0.001

The annual Previne Brasil Program prenatal indicator means did not reach the target set by the Ministry of Health, resulting in coefficients of 35.5% for the indicator that measures the minimum number and early prenatal consultations, and 50.1% for the indicator related to syphilis and HIV diagnostic tests for pregnant women.

Cluster 2 is made up of 444 municipalities, corresponding to 52.1% of the total municipalities in Minas Gerais. The congenital syphilis incidence rate was 5.1 cases/1,000 live births, significantly different to Cluster 1, but far from the rate recommended by the WHO. Regarding the Previne Brasil Program prenatal indicators, this group presented an average percentage above the target agreed upon by the policy, with 57.5% for the prenatal consultations indicator and 72.6% for the pregnant women undergoing syphilis and HIV tests indicator. Furthermore, it was the category with the largest stratum in terms of the number of PHC teams implemented and approved by the Ministry of Health, totaling 96.5% PHC team coverage.

Cluster 3 consists of 312 municipalities (36.6%), with a lower congenital syphilis incidence rate when compared to the other clusters (3.2 cases/1,000 live births). However, the variations in the two prenatal indicators were unfavorable in relation to the ideal value, with the lowest average percentage – namely, 25.7% for indicator 1 and 35.5% for indicator 2 – and, in turn, the PHC team coverage variable was 92.2%, remaining close to the PHC team coverage of the municipalities in Cluster 2. Cluster 3 comprises a group of municipalities that differs from the other clusters, that is, despite the high PHC team coverage, the prenatal indicators remained below the target recommended by the Ministry of Health. On the other hand, the congenital syphilis incidence remained at lower rates than in the other clusters.

**Figure 3:**
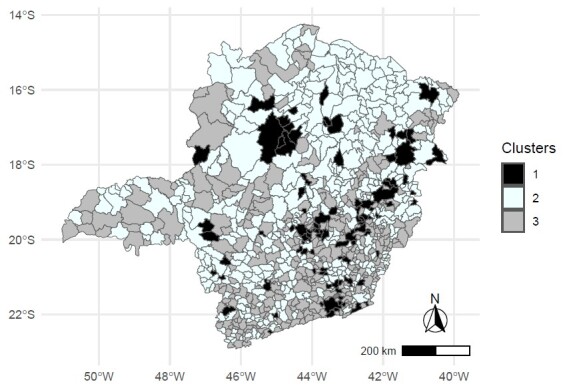
Spatial distribution of the Clusters according to congenital syphilis incidence, primary health care coverage, prenatal care indicator 1 (at least six consultations) and indicator 2 (testing for syphilis), by cluster. Minas Gerais, 2020-2022 (n=852)

Regarding spatial distribution (Figure 3), the map shows relevant geographic behavior between the clusters. The municipalities in Cluster 1 are distributed throughout the territory, not indicating significant concentration in certain health macro-regions. However, it is worth noting that some municipalities in the Center (20.8%), Vale do Aço (14.6%), Southeast (14.0%) and North (12.5%) health macro-regions stand out, compared to the other health macro-regions in Cluster 1.

In Cluster 2, it was possible to observe the predominance of municipalities that are under the jurisdiction of the North, Northeast and Jequitinhonha health macro-regions - 73.5% of the total municipalities in these health macro-regions are related to Cluster 2. The spatial clusters forming Cluster 3 indicated that 47.8% of the municipalities in this group are concentrated in the Southeast, Center, Far South and East-South health macro-regions, revealing important disparities between the Minas Gerais health macro-regions.

## Discussion

The results reveal an increasing trend in congenital syphilis in Minas Gerais, as well as the relationship between groups of municipalities with high PHC coverage and better prenatal indicators, with lower congenital syphilis incidence rates, especially in the health macro-regions in the North and Northeast of the state. However, use of secondary and aggregated data from information systems may have confounding factors, in addition to sociodemographic determinants, which may not accurately reflect local realities.

In the four-month periods comprising 2020 to 2022, there was an increase in congenital syphilis incidence in Minas Gerais, and, in the same period, there was a gradual increase in the performance of municipalities in relation to the minimum number of prenatal consultations begun early and syphilis and HIV testing, in addition to the increase in population coverage by PHC teams.

Analyses carried out in the states of Minas Gerais, Bahia and São Paulo found that the availability of prenatal consultations alone was not enough to mitigate cases of congenital syphilis, since there are late diagnoses and inadequate or non-existent treatments (13,14,15). In the United States, it was also found that 60.0% of cases of congenital syphilis in 2019 were due to inadequate treatment or failures in syphilis screening in pregnant women (16).

Analysis of the clusters revealed distinct behaviors between the groups of municipalities regarding congenital syphilis incidence and the role of PHC. The difference between Clusters 1 and 2 reinforces the strategic role of PHC in prevention, although access to and quality of prenatal care still represent significant challenges for controlling this condition in Minas Gerais.

In the United States, limitations in access to and timely prenatal care were observed in 28.0% of mothers with perinatal syphilis, which made it easier to understand the increase in cases of congenital syphilis in that country (17). In Europe, congenital syphilis rates higher than those recommended by the WHO were found, as a consequence of reduced or non-existent screening for syphilis during pregnancy (18).

Neglecting variables related to access to health services, linkage, and humanized care for interrupting vertical transmission of syphilis may lead to the risk of assessing the quality of prenatal care in isolation, based on the number of consultations and procedures provided to pregnant women, disregarding barriers to access and care provided by PHC professionals during pregnancy (19). 

In the state of Goiás, analysis covering the period from 2007 to 2014 showed an increase in congenital syphilis in municipalities with Family Health Strategy coverage below 75.0%, with no clear relationship between coverage above this level and reduction in rates (20). In Minas Gerais, between 2001 and 2018, regions with consolidated Family Health Strategy coverage showed a 24.4% reduction in congenital syphilis cases from 2010 onwards (21), highlighting disparities in relation to areas with low coverage.

The findings for Cluster 3 indicate gaps in the periodic follow-up and provision of tests for syphilis diagnosis in pregnant women by PHC teams; however, despite these weaknesses, congenital syphilis incidence in this group is lower. In this context, prenatal care may not have been effective and qualified, which may have hindered the detection of gestational diseases and neonatal infections, possibly due to insufficient maternal clinical and epidemiological history. Underreporting of congenital syphilis is a plausible hypothesis that deserves attention, especially in view of the deficiencies in the records, which may be influenced by a series of factors, ranging from accuracy of diagnosis to lack of prioritization in documenting information.

An ecological study conducted in the state of Espírito Santo pointed to the relationship between low syphilis incidence rates in pregnant women and congenital syphilis and underreporting, resulting from possible failures in care and lack of diagnosis (22). Likewise, in the South and Far South of the state of Bahia, similar behaviors were portrayed regarding the precariousness and nullity of congenital syphilis case reporting (23).

The spatial analysis showed a heterogeneous distribution of clusters, with some groups defined in certain health macro-regions, revealing geographic behaviors both in terms of care and epidemiological aspects in different areas of the state of Minas Gerais. A significant proportion of the municipalities in Cluster 1 were found to be concentrated in the Center and Vale do Aço health macro-regions. This observation echoes findings from previous studies, suggesting that this fact can be attributed to the high population flow in these areas, presence of densely populated municipalities, existence of industrial complexes and integration with metropolitan areas (22).

In the spatial comparison of Clusters 2 and 3, a geographic distinction was evident between the Northern and Southern regions of the state, indicating significant disparities in access to and care related to prenatal care. This differentiation reflects a healthcare and epidemiological reality that differs from the expected pattern between more or less economically developed regions. More developed areas, characterized by greater economic diversity, generally offer greater access to health goods and services, which, in turn, contribute to meeting health needs in a comprehensive manner and provide a more effective organizational structure (24).

Studies indicate that poorer regions in Minas Gerais performed better with regard to indicators of financial investment and access to public health, demonstrating budgetary efforts in these areas (25). In turn, in California (USA), vulnerable regions presented higher congenital syphilis incidence, in contrast to areas with better socioeconomic conditions (26). Differences in the health systems of the two countries may explain the divergent results.

In Juiz de Fora, a large municipality in Cluster 1, a study found that starting prenatal consultations early and the number of consultations recommended by ministerial programs, including Previne Brasil Program data, were unsatisfactory, increasing the outcomes related to vertical transmission of syphilis (27). In the city of Montes Claros, in the North health macro-region, forming part of Cluster 2, the majority of pregnant women achieved the minimum number of consultations and started them early, but even so the congenital syphilis incidence rates persisted in that territory (28).

In Belo Horizonte, the capital city of the state of Minas Gerais, 16.0% of mothers of children with congenital syphilis did not attend any prenatal consultations; in addition, diagnoses were made late, resulting in inadequate treatments and low detection of congenital syphilis, which corroborates the findings of our study (29).

The results of this study indicate that eliminating congenital syphilis remains a challenge for health services and their management, despite evidencing significant perceptions about the different care behaviors and congenital syphilis incidence in different health macro-regions and/or municipalities. It is essential to study further the role of PHC in communicable diseases, considering local-regional specificities. Including Previne Brasil Program indicators allowed us to assess the health care panorama in Minas Gerais, identifying the most vulnerable regions and pointing to ways to reduce inequalities and strengthen actions to address congenital syphilis.

## Data Availability

The data sets used in this research are available in the following repositories and platforms: Sistema e-Gestor AB – Relatórios Públicos: https://egestorab.saude.gov.br/paginas/acessoPublico/relatorios/relatoriosPublicos.xhtml. Minas Gerais State Health Department – TabNet: http://vigilancia.saude.mg.gov.br/index.php/informacoes-de-saude/informacoes-de-saude-tabnet-mg/. Universidade Federal de Uberlândia Repository: ELLER, Bruna Betiatti Benatatti. Incidência de sífilis congênita relacionada à cobertura de Atenção Primária à Saúde e do pré-natal no estado de Minas Gerais no ano de 2022. 2024. Available at: https://repositorio.ufu.br/handle/123456789/43107.
