# Supplementary material for: Congenital syphilis related to primary health care and prenatal care coverage: a spatial analysis, Minas Gerais, 2020-2022
Source: Epidemiol Serv Saude. 2025 Jul 11;34:e20240495. doi: 10.1590/S2237-96222025v34e20240495.en (PMC12266656; doi:10.1590/S2237-96222025v34e20240495.en)
Supplement: Translation

# Translation: Sífilis congênita relacionada à cobertura da atenção primária à saúde e do pré-natal: análise espacial, Minas Gerais, 2020-2022

**DOI:** 10.1590/S2237-96222025v34e20240495.pt

**Authors:** Bruna Betiatti Benatatti Eller, Marcelle Aparecida de Barros Junqueira, Lucio Borges de Araújo

**Affiliations:** 1 Universidade Federal de Uberlândia, Programa de Pós-Graduação em Saúde da Família, Uberlândia, MG, Brasil; 2 Universidade Federal de Uberlândia, Instituto de Matemática e Estatística, Uberlândia, MG, Brasil

**Keywords:** Sífilis Congênita, Cuidado Pré-Natal, Atenção Primária à Saúde, Programas Nacionais de Saúde, Sistemas de Informação em Saúde

## Abstract

**Objetivo:**

Analisar a variação das incidências da sífilis congênita, segundo os indicadores de pré-natal e cobertura da atenção primária à saúde (APS) em Minas Gerais, entre 2020 e 2022.

**Métodos:**

Análise espacial apresentada por meio de aglomerados, através da técnica não hierárquica k-means e do georreferenciamento espacial, com dados secundários do Sistema de Informação em Saúde para Atenção Básica e do Portal da Vigilância em Saúde da Secretaria de Estado de Minas Gerais.

**Resultados:**

O aglomerado 1 apresentou menores indicadores de pré-natal (35,5%) e cobertura da APS (88,6%), com elevadas incidências de sífilis congênita (36,5 casos/1 mil nascidos vivos). O aglomerado 2 demonstrou menores incidências da doença (5,1 casos/1 mil nascidos vivos) e melhor cobertura de APS (96,5%) e pré-natal (57,5%). O aglomerado 3 registrou baixo desempenho nos indicadores de pré-natal (25,7%), cobertura de APS adequada (92,2%), e incidências reduzidas da sífilis congênita (3,21 casos/1 mil nascidos vivos).

**Conclusão:**

Diferentes comportamentos assistenciais e das incidências de sífilis congênita foram identificados entre macrorregiões de saúde e/ou municípios de Minas Gerais; assim, o estudo poderá contribuir para a formulação de políticas públicas no enfrentamento dessa infecção.

**Aspectos éticos**

Esta pesquisa utilizou bancos de dados de domínio público e anonimizados.:

## Introdução

A sífilis é uma infecção sexualmente transmissível que, no período gestacional, pode causar, em até 40,0%, aumento nas proporções de mortes fetais, além das mortes neonatais. A transmissão da mãe para o feto pode ser reduzida em até 98,0% se o diagnóstico e o tratamento da sífilis em gestantes forem realizados oportunamente (1).

O cenário epidemiológico da sífilis congênita, em escala global, alcançou estimativas de 473 (IC95% 385; 561)/100 mil nascidos vivos em 2016, com destaque para o continente africano, que registrou 61,0% do total de casos (2). No Brasil, em 2021, as taxas de incidência de sífilis congênita atingiram 9,9/1 mil nascidos vivos (3).

A Organização Mundial da Saúde tem elaborado estratégias para a eliminação da sífilis congênita, salientando que as incidências correspondem a 0,5 ou menos casos por 1 mil nascimentos até 2030 (4). O papel estratégico da atenção primária à saúde (APS) na prevenção e na intervenção imediata em relação à sífilis gestacional é fundamental, pois é o local prioritário para a oferta dos cuidados prénatais, a ampliação de testes diagnósticos e o tratamento oportuno (5).

A qualidade do cuidado ofertado no pré-natal permanece como desafio nos serviços de saúde, em que as falhas assistenciais se associam ao ressurgimento desse agravo (6). A insuficiência da cobertura de saúde também é um dos pilares que facilmente contribuem para as barreiras estruturais do acesso e interfere nos cuidados pré-natais para as gestantes (7).

Em 2019, foi instituído um modelo de financiamento federal, o programa Previne Brasil, com novas propostas metodológicas de ação e avaliação da APS. Assim, foram atribuídos indicadores, nessa nova política, para direcionar e avaliar as ações ofertadas na APS – entre eles, indicadores de pré-natal, com a finalidade de assegurar abordagem precoce e acompanhamento contínuo, durante o período gestacional (8).

Há uma lacuna importante de estudos que relacionam a qualidade assistencial da APS às incidências da sífilis congênita, considerando-se o funcionamento e a organização da gestão entre os territórios. Este estudo teve como objetivo analisar a variação das taxas de incidência da sífilis congênita, conforme os indicadores assistenciais de pré-natal do Previne Brasil e de cobertura da APS, nos municípios de Minas Gerais, entre os anos de 2020 e 2022.

## Métodos

### Delineamento

Este estudo apresenta um delineamento de análise espacial, transversal e analítico, utilizando dados secundários de sistemas de informação em saúde pública para analisar a relação entre indicadores assistenciais de pré-natal e taxas de incidência de sífilis congênita em municípios de Minas Gerais, entre os anos de 2020 e 2022.

### Contexto

Foram utilizados dados secundários provenientes dos principais sistemas de informação do Sistema Único de Saúde (SUS): Sistema de Informação em Saúde para Atenção Básica e Portal da Vigilância em Saúde da Secretaria de Estado de Minas Gerais, de acesso público.

Minas Gerais conta com 28 Superintendências Regionais de Saúde, distribuídas em 12 mesorregiões, com uma população de aproximadamente 21 milhões de habitantes. A Região Metropolitana de Belo Horizonte, com mais de 5,5 milhões de habitantes, é o centro econômico e político do estado, com uma forte presença de serviços, comércio e indústria. A Central Mineira, com 2,5 milhões de habitantes, apresenta uma economia diversificada, com agricultura, pecuária e mineração.

As regiões do Norte de Minas e do Vale do Rio Doce têm economias baseadas na agricultura, na pecuária e na mineração, enquanto o Triângulo Mineiro/Alto Paranaíba é conhecido pelo agronegócio. A Zona da Mata é famosa pela produção de café. As mesorregiões do Campo das Vertentes, do Vale do Mucuri, do Jequitinhonha e do Alto Jequitinhonha apresentam desafios na infraestrutura de saúde em áreas rurais, com populações envelhecidas e dispersas.

### Dados populacionais

Inicialmente, a coleta dos dados foi realizada mediante acesso a informações sobre as incidências de sífilis congênita, a cobertura populacional pelas equipes da APS e os indicadores de pré-natal inseridos no programa Previne Brasil. Isso permitiu a estruturação de uma base de dados consistente para subsidiar a análise estatística deste estudo (9,10). 

### Variáveis

A taxa de incidência anual de sífilis congênita, acessada no Portal da Vigilância em Saúde da Secretaria de Estado de Minas Gerais, foi a principal variável da pesquisa, sendo mensurada para cada município através do número de casos novos notificados de sífilis congênita dividido pelo número de nascidos vivos no mesmo ano/local, multiplicado por mil. Os resultados percentuais dos indicadores de pré-natal constituíram as outras variáveis, identificados via Sistema de Informação da Atenção Básica. O indicador 1 avalia a proporção de gestantes com, no mínimo, seis consultas de pré-natal realizadas, sendo a primeira consulta até a 12ª de gestação. Por sua vez, o indicador 2 mensura a proporção de gestantes que realizaram exames de sífilis e vírus da imunodeficiência humana (HIV).

### Fontes de dados e mensuração

Para a construção dos cenários municipais com os indicadores de pré-natal, foram executadas as médias, separadamente, para o indicador 1 e, também, para o indicador 2, de acordo com os resultados alcançados no primeiro, segundo e terceiro quadrimestre de 2022 do programa Previne Brasil. Os parâmetros empregados para configurar a assistência ao pré-natal de qualidade foram igualmente preconizados como meta para a avaliação do desempenho das equipes da APS, fundamentadas pelo programa, sendo de 45,0% para o indicador 1 e de 60,0% para o indicador 2. A cobertura populacional pelas equipes de APS, também incluída no presente estudo como variável, foi obtida através da média anual de cobertura da APS. 

### Controle de viés

Para a avaliação da cobertura de APS nos municípios estudados, adotou-se como referencial a média estadual de cobertura de APS. O método de cálculo baseia-se no quantitativo da população cadastrada pela estratégia saúde da família (ESF) e pelas equipes de APS, em relação à população estimada pelo Instituto Brasileiro de Geografia e Estatística (IBGE) (11). 

A utilização de sistemas de informação em saúde como fonte de dados pode apresentar limitações, particularmente em contextos de emergências sanitárias, como a pandemia de covid-19. Entre os principais desafios, destacam-se a subnotificação, as inconsistências nos registros, as alterações nos fluxos de atendimento e a priorização de casos graves. Adicionalmente, a sobrecarga dos sistemas de saúde impactou negativamente a qualidade dos dados. Para minimizar esses vieses, foram implementadas estratégias como a validação cruzada com fontes secundárias, a análise de tendências temporais, ajustes estatísticos para correção de subnotificações e a exclusão de dados inconsistentes, garantindo-se, assim, a validade e a confiabilidade dos resultados obtidos no estudo.

### Tamanho do estudo

Foram extraídos e processados dados (taxa de incidência da sífilis congênita e médias dos indicadores da APS), quadrimestralmente, dos anos de 2020, 2021 e 2022, para a avaliação temporal comparativa, permitindo a compreensão robusta do cenário epidemiológico e assistencial de Minas Gerais.

### Métodos estatísticos

Na análise dos dados, foi empregada uma análise de aglomerados, uma abordagem estatística multivariada que organiza grupos distintos de municípios com base em suas características e similaridades, considerando as variáveis coletadas. A formação desses aglomerados combina municípios com alta homogeneidade interna e alta heterogeneidade externa, à medida que técnicas/funções estatísticas são adotadas com definições de medidas de proximidade e distância entre eles, através da medida de distância euclidiana (12).

Foi aplicada a técnica não hierárquica k-means, na qual os elementos são classificados a partir de uma definição prévia do número de agrupamentos e das estimativas iniciais dos centroides de cada aglomerado. Para evitar efeito de escala, as variáveis foram padronizadas antes da formação dos aglomerados. 

### Acesso aos dados e métodos de limpeza

Diversas configurações de agrupamentos foram testadas, e três agrupamentos foram escolhidos, considerando-se a interpretação teórica e a capacidade de discriminação dos componentes de cada grupo. O município de Piau foi identificado como um caso atípico, devido às dimensões estatísticas discrepantes em relação aos demais registros. Após a análise, esse caso foi excluído do banco de dados utilizado para a formação dos aglomerados, a fim de garantir a representatividade e a coerência das variáveis estudadas. Essa exclusão preventiva visa assegurar que os resultados da análise de aglomerados não sejam influenciados por valores atípicos, permitindo uma representação mais precisa e confiável dos padrões e das estruturas subjacentes nos dados.

### Pareamento dos dados

A contribuição das variáveis para a formação dos aglomerados foi avaliada pela técnica da análise de variância (ANOVA). Para cada aglomerado, foram estimadas a média, o desvio-padrão e a frequência de agrupamento. A diferença entre os aglomerados foi considerada estatisticamente significativa para cada variável, caso apresentasse p-valor>0,05. As análises foram realizadas utilizando-se o software JAMOVI v. 2.3.28. Para caracterizar e discriminar a variabilidade regional entre os aglomerados, foi elaborado o georreferenciamento espacial dos municípios de Minas Gerais, no software R v. 4.3.3., de modo que fosse possível identificar no mapa em qual aglomerado o município havia sido alocado. 

## Resultados

Na análise exploratória, abrangendo o período de 2020 a 2022, Minas Gerais revelou um pico de incidência de sífilis congênita de 13,8 casos/1 mil nascidos vivos no 2º quadrimestre de 2022, e aumento de 26,0% de casos, quando comparado com os valores registrados no início e no término dos quadrimestres desse período. Nos indicadores 1 e 2 de pré-natal, foi observada a evolução positiva dos resultados no decorrer do 1^o^ quadrimestre de 2020 até o 3^o^ quadrimestre de 2022. 

Ambos os indicadores evidenciaram melhorias, embora, no 3º quadrimestre de 2021, o indicador relacionado às consultas de pré-natal tenha registrado resultado de 40,0%, seguido por redução nos períodos subsequentes. No 3^o^ quadrimestre de 2022, os percentuais alcançados foram de 38,0% para o indicador 1 e de 52,0% para o indicador 2. No período de 2020 a 2022, Minas Gerais, a cada quadrimestre, registrou incremento no número de equipes de ESF e de APS, com média anual de aumento de 5,0%, culminando em uma cobertura de APS de 88,0% no 3º quadrimestre de 2022 (Figura 1).

**Figura 1:** Incidência da sífilis congênita, média dos indicadores de pré-natal e cobertura da atenção primária à saúde, nos quadrimestres. Minas Gerais, 2020-2022 (n=852)

No modelo da análise de aglomerados (Figura 2), no ano de 2022, a configuração dos três aglomerados revelou características distintivas, considerando-se as combinações dos indicadores analisados, em que o perfil dos municípios se estruturara da seguinte maneira:

**Figura 2:** Médias da taxa de incidência de sífilis congênita, cobertura de atenção primária à saúde, indicador 1 (mínimo de seis consultas) e 2 (exames de sífilis) de pré-natal, por aglomerados. Minas Gerais, 2020-2022 (n=852)

- Aglomerado 1: foi composto de municípios que evidenciaram limitações simultâneas, tanto no baixo desempenho assistencial relacionado aos indicadores de pré-natal e de cobertura da APS, como nas taxas de incidência de sífilis congênita, que se apresentaram elevadas.
- Aglomerado 2: abrangeu municípios com melhores indicadores de cobertura de APS e pré-natal, e menores taxas de sífilis congênita.
- Aglomerado 3: englobou municípios que registraram baixas taxas de incidência de sífilis congênita, porém com menores indicadores de pré-natal e cobertura alta de APS, em comparação ao aglomerado 2.

De forma detalhada (Tabela 1), o aglomerado 1 é representado por 96 municípios, configurando 11,2% do total de municípios de Minas Gerais. Com relação às taxas da sífilis congênita, foi o aglomerado com a maior média de incidência da infecção congênita (36,5 casos/1 mil nascidos vivos), sendo superior aos aglomerados 2 e 3. Nesse mesmo grupo, os índices da cobertura de equipes da APS se apresentaram com valor médio inferior aos demais aglomerados, correspondendo a 88,6%.

**Tabela 1:** Quantidade de municípios, médias (%), desvio-padrão (DP) da taxa de incidência de sífilis congênita, cobertura de atenção primária à saúde, indicador 1 (mínimo de seis consultas) e 2 (realização de sífilis) de pré-natal, por aglomerados. Minas Gerais, 2020-2022 (n=852)

Aglomerado	Quantidade de municípios	Taxa de incidência de sífilis congênita % (DP)	Cobertura da atenção primária à saúde % (DP)	Indicador 1 – consultas de pré-natal % (DP)	Indicador 2 – exames de sífilis e HIV % (DP)
1	96	36,5 (11,7)	88,6 (15,6)	35,5 (18,2)	50,1 (21,3)
2	444	5,1 (7,7)	96,5 (6,8)	57,5 (12,4)	72,6 (13,9)
3	312	3,2 (5,4)	92,2 (11,7)	25,7 (14,7)	35,5 (17,8)
p-valor		<0,001	<0,001	<0,001	<0,001

A média anual dos indicadores de pré-natal do programa Previne Brasil não alcançou a meta estipulada pelo Ministério da Saúde, resultando em coeficientes de 35,5% para o indicador que mensura o número mínimo e precoce de consultas de pré-natal, e 50,1% para o indicador relacionado a testes diagnósticos de sífilis e HIV nas gestantes.

O aglomerado 2 é formado por 444 municípios, correspondendo a 52,1% do total de Minas Gerais. As taxas de incidência de sífilis congênita foram de 5,1 casos/1 mil nascidos vivos, com discrepância significativa do aglomerado 1, porém distante do valor recomendado pela OMS. Quanto aos indicadores de pré-natal do programa Previne Brasil, esse grupo apresentou percentual médio acima da meta pactuada pela política, sendo 57,5% para o indicador de consultas de pré-natal e 72,6% para gestantes com realização de exames para sífilis e HIV. Ademais, foi a categoria com o maior estrato em número de equipes de APS implantadas e homologadas pelo Ministério da Saúde, perfazendo um quantitativo de 96,5% da cobertura de equipes de APS.

Com relação ao aglomerado 3, ele é constituído de 312 municípios (36,6%), com taxa de incidência de sífilis congênita inferior quando se compara com os demais aglomerados (3,2 casos/1 mil nascidos vivos). Entretanto, as variações nos dois indicadores de pré-natal foram desfavoráveis no que se refere ao valor ideal, tendo o menor percentual médio – a saber, 25,7% para o indicador 1 e 35,5% para o indicador 2 –, e, por sua vez, a variável de cobertura de equipes da APS se apresentou em 92,2%, permanecendo próxima às coberturas de equipes da APS dos municípios pertencentes ao aglomerado 2. O aglomerado 3 compreende um grupo de municípios que se diferencia dos demais aglomerados, isto é, apesar da cobertura de equipes de APS estar elevada, os indicadores de pré-natal se mantiveram abaixo da meta preconizada pelo Ministério da Saúde. Por outro lado, as incidências da sífilis congênita permaneceram com taxas inferiores aos demais aglomerados.

Quanto à distribuição espacial (Figura 3), o mapa evidencia comportamento geográfico relevante entre os aglomerados. Os municípios pertencentes ao aglomerado 1 se encontram em disposição difusa no território, não indicando concentração expressiva em determinadas macrorregiões de saúde. Porém, cabe apontar que alguns municípios das macrorregiões de saúde do Centro (20,8%), do Vale do Aço (14,6%), do Sudeste (14,0%) e do Norte (12,5%) se destacam, em comparação às outras macrorregiões de saúde do aglomerado 1.

**Figura 3:** Distribuição espacial dos Aglomerados segundo as variáveis de incidência de sífilis congênita, cobertura de atenção primária à saúde, indicador 1 (mínimo de seis consultas) e 2 (realização de sífilis) de pré-natal, por aglomerado. Minas Gerais, 2020-2022 (n=852)

No aglomerado 2, foi possível observar a predominância dos municípios que estão sob a jurisdição das macrorregiões de saúde Norte, Nordeste e Jequitinhonha – 73,5% do total dos municípios dessas macrorregiões de saúde estão relacionados ao aglomerado 2. A relação dos aglomerados espaciais do aglomerado 3 apontou que 47,8% dos municípios desse grupo estão concentrados nas macrorregiões de saúde do Sudeste, Centro, Extremo Sul e Leste-Sul, revelando disparidades importantes entre as macrorregiões de saúde de Minas Gerais.

## Discussão

Os resultados revelam comportamento crescente da sífilis congênita em Minas Gerais e a relação entre os grupos de municípios com altas coberturas de APS e melhores indicadores de pré-natal, com menores incidências de sífilis congênita, especialmente nas macrorregiões de saúde do Norte e Nordeste do estado. Porém, a utilização de dados secundários e agregados oriundos de sistemas de informação pode dispor de fatores confundidores, além dos determinantes sociodemográficos, os quais podem não refletir de maneira precisa as realidades locais. 

Nos quadrimestres de 2020 a 2022, evidenciou-se um aumento nas incidências de sífilis congênita em Minas Gerais, e, nesse mesmo período, ocorreu um aumento gradativo no desempenho dos municípios em relação ao número mínimo de consultas de pré-natal com início precoce e à realização de exames de sífilis e o HIV, além do incremento das coberturas populacionais pelas equipes de APS.

Análises realizadas em Minas Gerais, Bahia e São Paulo constataram que somente a oferta de consultas pré-natais não era suficiente para mitigar os casos de sífilis congênita, uma vez que há diagnósticos tardios e tratamentos inadequados ou inexistentes (13,14,15). Em 2019, também foi observado que 60,0% dos casos de sífilis congênita derivaram de tratamento inadequado ou falhas no rastreamento da sífilis em gestantes nos Estados Unidos (16). 

A análise dos aglomerados revelou comportamentos distintos entre os grupos de municípios quanto às incidências de sífilis congênita e à atuação da APS. A diferença entre os aglomerados 1 e 2 reforça o papel estratégico da APS na prevenção, embora o acesso e a qualidade do pré-natal ainda representem desafios significativos para o controle desse agravo em Minas Gerais.

Nos Estados Unidos, as limitações no acesso e nos cuidados pré-natais, de forma oportuna, foram observadas em 28,0% das mães com sífilis perinatal, o que facilitou compreender o incremento dos casos de sífilis congênita no país (17). Na Europa, foram observadas taxas de sífilis congênita superiores ao valor recomendado pela OMS, como consequência da redução ou da inexistência do rastreamento para sífilis durante a gestação (18).

Negligenciar variáveis de acesso aos serviços de saúde, vínculo e acolhimento humanizado para a interrupção vertical da sífilis pode levar ao risco de avaliar isoladamente a qualidade do pré-natal, com base no número de consultas e procedimentos dispensados às gestantes, desconsiderando-se as barreiras de acesso e do acolhimento prestados pelos profissionais da APS nos cuidados durante a gestação (19).

Em Goiás, uma análise entre 2007 e 2014 mostrou aumento da sífilis congênita em municípios com cobertura da ESF abaixo de 75,0%, sem relação clara entre cobertura acima desse índice e redução das taxas (20). Em Minas Gerais, entre 2001 e 2018, regiões com cobertura consolidada da ESF apresentaram reduções de 24,4% nos casos de sífilis congênita a partir de 2010 (21), evidenciando disparidades em relação a áreas com baixa cobertura.

Os achados do aglomerado 3 indicam lacunas no acompanhamento periódico e na oferta dos exames para o diagnóstico da sífilis em gestantes pelas equipes da APS; no entanto, apesar dessas fragilidades, a incidência da sífilis congênita nesse grupo é menor. Nesse contexto, talvez, os cuidados pré-natais não tenham sido efetivos e qualificados, o que pode ter dificultado a detecção para as doenças gestacionais e as infecções neonatais, possivelmente pela insuficiência da história clínico-epidemiológica materna. A subnotificação da sífilis congênita é uma hipótese plausível que merece atenção, especialmente em vista das deficiências nos registros, que podem ser influenciadas por uma série de fatores, desde a precisão do diagnóstico até a falta de priorização na documentação.

Estudo ecológico realizado no Espírito Santo apontou a relação das baixas taxas de incidência de sífilis em gestantes e sífilis congênita com a subnotificação, decorrente de possíveis falhas na assistência e ausência de diagnósticos (22). Da mesma forma, no Sul e Extremo Sul da Bahia, foram retratados comportamentos semelhantes quanto à precariedade e à nulidade das notificações da sífilis congênita (23).

Na análise espacial realizada, houve distribuição heterogênea dos aglomerados, com alguns grupos definidos em determinadas macrorregiões de saúde, revelando comportamentos geográficos tanto assistenciais quanto epidemiológicos em diferentes áreas do estado de Minas Gerais. Foi encontrada proporção significativa dos municípios pertencentes ao aglomerado 1 concentrada nas macrorregiões de saúde do Centro e do Vale do Aço. Essa observação repercute descobertas de estudos anteriores, sugerindo que esse fato pode ser atribuído ao elevado fluxo populacional nessas áreas, à presença de municípios densamente povoados, à existência de complexos industriais e à integração com áreas metropolitanas (22).

Na comparação espacial dos aglomerados 2 e 3, evidenciou-se uma distinção geográfica entre as regiões Norte e Sul do estado, sinalizando disparidades significativas no que concerne ao acesso e aos cuidados relacionados ao pré-natal. Essa diferenciação reflete uma realidade assistencial e epidemiológica que diverge do padrão esperado entre as regiões mais ou menos desenvolvidas economicamente. As áreas mais desenvolvidas, caracterizadas por uma maior diversidade econômica, geralmente oferecem maior acesso a bens e serviços de saúde, que, por sua vez, contribuem para atender às necessidades de saúde, de forma abrangente, e proporcionam uma estrutura organizacional mais eficaz (24).

Estudos apontam que regiões mais carentes em Minas Gerais apresentaram melhor desempenho em indicadores de investimento financeiro e acesso à saúde pública, demonstrando esforços orçamentários nessas áreas (25). Por seu turno, na Califórnia, regiões vulneráveis apresentaram maior incidência de sífilis congênita, em contraste com áreas de melhores condições socioeconômicas (26). As diferenças nos sistemas de saúde dos dois países podem explicar os resultados divergentes.

Em Juiz de Fora, município pertencente ao aglomerado 1 e de grande porte, um estudo identificou que o início precoce e o número de consultas preconizadas pelos programas ministeriais, incluindo os dados do Previne Brasil, foram insatisfatórios, ampliando os desfechos relacionados à transmissão vertical da sífilis (27). Em Montes Claros, na macrorregião de saúde do Norte e pertencente ao aglomerado 2, a maioria das gestantes alcançou o mínimo de consultas e com início precoce, porém as incidências de sífilis congênita persistiram no território (28).

Em Belo Horizonte, 16,0% das mães de crianças com sífilis congênita não realizaram nenhuma consulta de pré-natal; além disso, os diagnósticos foram produzidos tardiamente, resultando em tratamentos inadequados e baixa detecção de sífilis congênita, o que corrobora os achados do estudo (29).

Os resultados deste estudo apontam que a eliminação da sífilis congênita permanece como desafio para os serviços de saúde e as instâncias gestoras, apesar de evidenciar percepções significativas sobre os diferentes comportamentos assistenciais e as incidências de sífilis congênita em diferentes macrorregiões de saúde e/ou municípios. É essencial aprofundar os estudos sobre o papel da APS nas doenças transmissíveis, considerando-se as especificidades locorregionais. A inclusão dos indicadores do programa Previne Brasil permitiu avaliar o panorama assistencial em Minas Gerais, identificando regiões mais vulneráveis e apontando caminhos para reduzir desigualdades e fortalecer ações no combate à sífilis congênita.

## Data Availability

Os conjuntos de dados utilizados na pesquisa estão disponíveis nos seguintes repositórios e plataformas: Sistema e-Gestor AB – Relatórios Públicos: https://egestorab.saude.gov.br/paginas/acessoPublico/relatorios/relatoriosPublicos.xhtml. Secretaria de Estado da Saúde de Minas Gerais – TabNet: http://vigilancia.saude.mg.gov.br/index.php/informacoes-de-saude/informacoes-de-saude-tabnet-mg/. Repositório Institucional da Universidade Federal de Uberlândia: ELLER, Bruna Betiatti Benatatti. Incidência de sífilis congênita relacionada à cobertura de Atenção Primária à Saúde e do pré-natal no estado de Minas Gerais no ano de 2022. 2024. Disponível em: https://repositorio.ufu.br/handle/123456789/43107.
